# Current Trends in Metallic Orthopedic Biomaterials: From Additive Manufacturing to Bio-Functionalization, Infection Prevention, and Beyond

**DOI:** 10.3390/ijms19092684

**Published:** 2018-09-10

**Authors:** Amir A. Zadpoor

**Affiliations:** Additive Manufacturing Laboratory, Department of Biomechanical Engineering, Faculty of Mechanical, Maritime, and Materials Engineering, Delft University of Technology (TU Delft), Delft 2628CD, The Netherlands; a.a.zadpoor@tudelft.nl; Tel.: +31-15-278-1051

**Keywords:** metallic biomaterials, orthopedic applications, additive manufacturing, surface bio-functionalization, implant-associated infections, biodegradable metals, composite metallic biomaterials

## Abstract

There has been a growing interest in metallic biomaterials during the last five years, as recent developments in additive manufacturing (=3D printing), surface bio-functionalization techniques, infection prevention strategies, biodegradable metallic biomaterials, and composite biomaterials have provided many possibilities to develop biomaterials and medical devices with unprecedented combinations of favorable properties and advanced functionalities. Moreover, development of biomaterials is no longer separated from the other branches of biomedical engineering, particularly tissue biomechanics, musculoskeletal dynamics, and image processing aspects of skeletal radiology. In this editorial, I will discuss all the above-mentioned topics, as they constitute some of the most important trends of research on metallic biomaterials. This editorial will, therefore, serve as a foreword to the papers appearing in a special issue covering the current trends in metallic biomaterials.

## 1. Introduction

The research on metallic biomaterials has recently enjoyed a surge in interest, as specific developments, particularly maturation of additive manufacturing (=3D printing) techniques, have provided a host of new possibilities. Due to their high mechanical properties, metallic biomaterials have been used for applications in which load-bearing capability is the major deciding factor. Skeletal applications have, therefore, been the main areas of interest, as the vast majority of orthopedic implants are made from metals, at least partially.

Similar to other types of biomaterials, the most important aim in the development of metallic biomaterials is to achieve multiple advanced functionalities. In the case of orthopedic implants, those advanced functionalities should ultimately result in improved longevity of permanent implants, enhanced tissue regeneration performance of bone substitutes, more effective reconstruction of large bony defects, and minimized risk of implant-associated infections. The current research trends on metallic biomaterials are, therefore, focused on achieving the above-mentioned goals, and include 1. Application of advanced additive manufacturing techniques to design and fabricate biomaterials with favorable properties and performance; 2. Bio-functionalized surfaces that could improve bone regeneration performance while minimizing the risk of implant-associated infections; 3. Introducing biodegradation into metallic bone substitutes; and 4. Development of composite biomaterials where metallic biomaterials are combined with ceramic or polymeric materials. Every one of these trends will be discussed in the following sections of this editorial. Another important trend that has emerged during the last few years is application of the techniques developed in the other branches of biomedical engineering, particularly tissue biomechanics, musculoskeletal dynamics, and skeletal radiology, to rationally design metallic biomaterials with better functionalities. We will discuss the relationship between metallic biomaterials and the related areas of biomedical engineering research in the last section of this editorial. As a whole, this editorial aims to present a global picture of some of the most important research trends in the metallic orthopedic biomaterials community. This should provide the context for the papers that follow in this special issue of the International Journal of Molecular Sciences, titled “Current Trends in Metallic Biomaterials: From Additive Manufacturing to Bio-Functionalization, Infection Prevention, and Beyond”.

## 2. Additive Manufacturing

Metal 3D printing, otherwise known as metal additive manufacturing, has emerged during the last decade as one of the most successful types of printing techniques for fabrication of functional medical devices. Prior to the advent and maturation of additive manufacturing, the research into metallic biomaterials had become somewhat stagnant, as early developments had made their way into clinical applications, while the possibilities for further developments seemed somewhat limited. Consequently, many research groups were focusing on polymeric and ceramic biomaterials. The tremendous success of metal printing has, however, changed the research landscape since then. In particular, additive manufacturing presents a unique opportunity for adjusting the mechanical properties of metallic biomaterials using topologically ordered volume-porous biomaterials. The mechanical properties of the metallic alloys used for fabrication of orthopedic implants are usually much higher than those of the native bone tissue, increasing the risk of stress shielding [1,2,3,4]. The mechanical properties could, however, be adjusted to the level of the native bone tissue using topologically ordered volume-porous designs. Since porous designs could only achieve mechanical properties that are below those of the base material, they do not help in increasing the mechanical properties of the materials whose mechanical properties are lower than those of the native bone tissue (e.g., most polymeric materials). This provides a significant advantage for metallic biomaterials, as they could now provide any practically relevant set of mechanical properties, including the values comparable with those of trabecular bone (i.e., *E* < 1–2 GPa), to values in the range of those observed for cortical bone (i.e., 1 < *E* < 15 GPa) and beyond [5,6,7,8].

In addition to adjusting the mechanical properties, topologically ordered volume-porous metallic biomaterials could be used for adjusting the other types of properties that are important for their application as orthopedic implants and bone substitutes. That includes fluid flow and mass transport properties (e.g., permeability and diffusivity) [9,10,11] and curvature [12,13,14]. It is therefore possible to create metallic biomaterials with unique and unprecedented combinations of properties favorable for bone tissue regeneration and bony ingrowth. This gives rise to the topics of “design for additive bio-manufacturing” and “meta-biomaterials”, that are discussed at length in one of the review papers appearing in the current special issue [15]. In short, meta-biomaterials [5,9,10,11,16] use the topological design at the microscale to create biomaterials with unique combinations of quasi-static mechanical properties [8,17], permeability [9,10], fatigue resistance [18,19,20,21], and biological behavior. Establishing topology–property relationships using analytical [22,23,24] and computational [25,26] models is, therefore, an important aspect when designing meta-biomaterials.

Moreover, additive manufacturing techniques have rendered fully patient-specific implants as a feasible design option (see also Section 6. Interfacing with Biomechanics and Image Processing). Patient-specific implants may be wholly or partially based on porous biomaterials, but that is not a requirement, and other designs may work better for some applications. Moreover, the patient-specific aspect could be extended to cover the design of the microscale topology that is used for inducing specific combinations of favorable properties.

Finally, additive manufacturing has fueled the progress in other areas of research on metallic biomaterials through introduction of topologically ordered volume-porous biomaterials. Volume-porous biomaterials possess two important features, namely huge surface areas and large pore spaces. These features could be helpful for bio-functionalization and infection prevention through techniques that either modify the biomaterial surfaces or leverage drug delivery vehicles that could be efficiently accommodated in the available pore space.

## 3. Bio-Functionalization

Bio-functionalization constitutes an important frontier of research on metallic biomaterials. Although bio-functionalization may refer to all types of bio-functionalities, including improved tissue regeneration performance, infection prevention, and modulation of the immune response, we focus, here, on the tissue regeneration performance, as infection prevention is covered in the next section, and the research into other types of functionalities have, in most cases, only started recently.

The bio-functionalization techniques aim for improvement of the tissue regeneration performance and bony ingrowth of metallic biomaterials, and may work through (bio)chemical or physical mechanisms. The techniques based on chemical and biochemical agents have been most extensively researched. In particular, it has been shown that the local delivery of specific growth factors, such as bone morphogenic protein (BMP), could result in improved tissue regeneration performance [27,28,29]. Moreover, the local delivery of specific elements, such as strontium, have been shown to increase bone regeneration performance [30].

On the physical side, recent discoveries have shown that stem cell fate could be determined with nanoscale topographies present on the surface of biomaterials [31,32,33]. The mechanobiological mechanisms governing this response are very different from those of the above-mentioned (bio)chemical compounds, and are primarily related to the mechanical forces sensed by the stem cells and the adjustment of focal adhesions [34,35]. There is a growing body of literature showing that osteogenic differentiation could be stimulated using surface nanoscale topographies with feature sizes below 100 nm [36]. Determining the best shape and size of nanoscale features, however, remains an important area of research, with many potential applications. Furthermore, development of nano-fabrication techniques, that allow for decorating the surface of 3D printed biomaterials with such nanoscale topographies, is technologically challenging, particularly when trying to apply nano-topographical features to topologically ordered volume-porous biomaterials. A combination of origami techniques and self-folding techniques [37] has been recently proposed as a possible way to approach this problem, by folding the porous biomaterials from a flat surface. The research into foldable biomaterials is, nevertheless, at its fancy, and requires many further developments.

Ultimately, it may be important to combine the (bio)chemical approach with the physical one to maximize the tissue regeneration performance of metallic biomaterials while minimizing the risk of drug-related toxicities. This line of research has not received much attention, as of yet, and could be a worthy research avenue for future studies.

Several studies appearing in this special issue address bio-functionalization, biocompatibility, and bioactivity of (metallic) biomaterials [38,39,40,41,42]. Some other surface-related have been addressed as well, e.g., [43].

## 4. Infection Prevention

Prevention and treatment of implant-associated and biomaterial-associated infections are one of the priorities in orthopedic surgeries. This is particularly important for the most vulnerable patient groups, such as orthopedic oncologic patients whose immune system may have been compromised due to chemotherapy and/or radiotherapy, or trauma patients who have to undergo a long surgery with a large open wound that is needed for implantation of large implants. In many such cases, metallic biomaterials that minimize the risk of infections could potentially play an important role.

Similar to the case of bio-functionalization techniques aimed towards improvement of bone tissue regeneration performance, the biomaterials that minimize the risk of infection may work on the basis of (bio)chemical antibacterial agents or physical mechanisms. The (bio)chemical approaches are usually based on antibiotics [44], antimicrobial peptides [45], or inorganic (mostly metallic) antibacterial agents, such as silver [46,47,48,49], zinc [50,51], or copper [52,53]. Moreover, recent research suggests that simultaneous (local) delivery of antibiotics and inorganic antibacterial agents could give rise to synergistic effects [54,55].

On the physical side, nanoscale patterns, including those with large aspect ratios, are found to not only affect bacterial adhesion to the surfaces, but also kill bacteria [56]. Moreover, a number of natural surfaces, such as dragonfly wing and cicada wings, that are covered by such types of nanopatterns, clearly show antibacterial behavior [57,58]. Mechanical forces and excessive deformations are proposed as possible mechanisms through which nanopatterns kill bacteria [59]. In that sense, the physical mechanisms of infection prevention are similar to those affecting stem cell fate. It may, therefore, be possible to combine both types of functionalities into the same types of biomaterials [60]. Moreover, efficient fabrication of the nanopatterns on metallic surfaces is challenging, for which more efficient and precise techniques need to be developed.

## 5. Biodegradable Metallic Biomaterials

Although biodegradable metallic biomaterials have been under development for quite some time [61,62,63,64] and are addressed in this special issue as well [65], fabricating biodegradable metallic biomaterials with a fully interconnected porosity and topologically ordered design has not been possible until very recently [66,67] where direct metal printing has enabled fabrication of such materials. The number of materials from which biodegradable porous metallic biomaterials have been made with additive manufacturing are, however, still very limited, and a lot of further research is required for advancing the field. Moreover, the long-term in vivo effects of biodegradable metals are not currently well understood. More research is therefore needed to clarify what the local and systemic effects of biodegradable biomaterials are, to determine the size distribution of the biodegradation products, to understand how the biodegradation products interact with the immune system, and to evaluate the cytocompatibility of the biodegradable metals in vivo.

Similar to the case of mechanical properties, the biodegradation profile, as well as the characteristics of the biodegradation products, could potentially be adjusted using the topological design at the microscale. In addition, the large surface area of topologically ordered volume-porous biomaterials could be used to speed up the biodegradation process, thereby facilitating the application of metals that degrade too slowly, e.g., zinc or iron. The relationship between the microscale topological design and biodegradation profile, however, remains largely elusive.

## 6. Interfacing with Biomechanics and Image Processing

Design of new (meta-)biomaterials with novel properties requires application of techniques developed in the other branches of biomedical engineering and skeletal radiology. Two particular types of expertise are notable in this regard, namely, biomechanical modeling and medical image processing.

The biomechanical models used in orthopedic biomechanics could be divided into two main subcategories: 1. Tissue biomechanics and 2. Musculoskeletal dynamics. Tissue biomechanics modeling allows for characterization and study of the mechanical and physical (e.g., mass transport) behaviors of biological tissues. Since mimicking the properties of the native tissue is often considered the ultimate goal of the biomaterials that are used to replace them either temporarily or permanently, this type of characterization is crucial to the design of new biomaterials. In the vast majority of cases, tissue biomechanics models [68,69,70,71] are basically computational mechanics (e.g., finite element) models of the tissue at different length scales. One of the most important input data for creating such tissue biomechanics models is the actual musculoskeletal loading conditions. Musculoskeletal dynamics models enter the picture at this stage, as they could provide estimations of the musculoskeletal loading conditions with varying degrees of accuracy. Among different types of musculoskeletal dynamics models, large-scale musculoskeletal models [72,73,74] are, in principle, more accurate than mass–spring–damper models [75,76], and provide more detailed loading estimations. However, large-scale musculoskeletal models require many more parameters and input data, and are more difficult to work with.

Image processing techniques are required to obtain the shapes of the bones and other skeletal tissues at different scales. Moreover, tissues with different (mineral) densities attenuate the X-ray beams differently, thereby allowing use of the gray scale data of the resulting images as a way to estimate the distribution of the tissue density. Combining the data regarding the shape and density distribution of bones is crucial for building computational tissue biomechanics models discussed above. Moreover, advanced image processing techniques, such as those based on statistical shape models and statistical shape and appearance models [77] could facilitate the process of shape and density analysis and interfacing imaging data with both tissue biomechanics and musculoskeletal dynamics models.

Ideally, one would like to combine image processing techniques with musculoskeletal dynamics models and tissue biomechanics models, to develop a streamlined approach for design of meta-biomaterials based on quantitative data regarding the shape, density distribution, loading conditions, micro-architectural design, and mechanical properties of tissues and organs. The same type of data could be used for designing patient-specific implants and evaluating the designed implants to see whether the resulting stress/strain distributions are comparable with those observed in the native tissues. This integrated approach is a relatively new line of research, particularly in connection with the design of biomaterials, and could result in some new ways of designing biomaterials and implants that are based on physical models and quantitative data, as opposed to the heuristic approaches used in the past. One of the review papers appearing in this special issue addresses some of the above-mentioned topics [78].

## 7. Composite Biomaterials

Even though metallic biomaterials offer a host of favorable properties whose ranges could be further expanded using rational design techniques and additive manufacturing, other categories of biomaterials, including ceramics and polymers, could add additional functionalities or broaden the possible ranges of properties. That is why composite biomaterials combining different categories of biomaterials could be of great interest and value. The research into this category of biomaterials has been ongoing for several years (see e.g., [79]). However, new horizons are appearing given an ever-increasing number of polymeric, ceramic, and metallic materials that could be combined with each other. Moreover, new processing techniques including multimaterial additive manufacturing techniques and post-additive manufacturing techniques, could open up many new opportunities for combining materials from different categories with specific microscale topological designs and spatial distributions to achieve novel functionalities and properties. This is a promising area of future research that could particularly benefit from continuing advances in additive manufacturing technologies. Some early adoptions of this line of research (composites) appear in this special issue [80], but more research is required to realize the full potential of this approach.

## 8. Conclusions

We briefly reviewed some of the current trends of research on metallic biomaterials, particularly the metallic biomaterials aimed for skeletal applications, such as permanent orthopedic implants or the temporary bone substitutes used for treatment of (large) bony defects. The topics identified here are currently being intensively researched, and could lead to breakthroughs in treatment of skeletal diseases. The relevant literature, including the papers published in this special issue as well as those published elsewhere, were cited. Following up on the research areas suggested here, with new ideas and techniques, could further stimulate the success of metallic biomaterials community as a whole, while offering new generations of metallic medical devices.
